# Frequency of placenta previa in previously scarred and non scarred uterus

**DOI:** 10.12669/pjms.312.6509

**Published:** 2015

**Authors:** Tayyaba Majeed, Fatima Waheed, Zahid Mahmood, Kanwal Saba, Hamis Mahmood, Mulazim Hussain Bukhari

**Affiliations:** 1 Tayyaba Majeed, MBBS, FCPS. Associate Professor of Gynae and Obst., Lady Aitchicen Hospital, Lahore, Pakistan; 2 Fatima Waheed, MBBS, FCPS. Lady Willingdon Hospital, Lahore, Pakistan; 3 Zahid Mahmood, Lahore Medical and Dental College, Lahore, Pakistan; 4 Kanwal Saba, MBBS, MSC, MPhil. Department of Pathology, King Edward Medical University, Lahore, Pakistan; 5 Hamis Mahmood, BCS. Computer Specialist, Lady Aitchicen Hospital, Lahore, Pakistan; 6 Mulazim Hussain Bukhari, MBBS, DCP, MPhil, FCPS, PhD. Department of Pathology, King Edward Medical University, Lahore, Pakistan

**Keywords:** Placenta Previa, Frequency, Previously scarred and non-scarred uterus

## Abstract

**Objective:**

To determine the frequency of placenta Previa in patients coming to a tertiary care unit with previously scarred and non-scarred uterus.

**Methods:**

A descriptive cross sectional study was carried on 114 cases who underwent caesarean sections (37 cases out of 645 cases with non scarred uterus and 77 cases from 721 cases with scarred uterus) in the department of obstetrics and gynecology Lady Willingdon Hospital from January 2008– December 2011.

**Results:**

Most patients (47.36%) were between 26-30 years age group, presented with gestational age between 36-40 weeks (70.17%), were mostly G2-4, while frequency of placenta Previa in non-scarred uterus was 32.45% (37 cases), and frequency in previously scarred uterus was 67.54% (77 cases). Major degree Previa was found in 88 cases (77.19%). There were 5.70% cases of placenta Previa from non-scarred uteruses and 10.67% cases of placenta Previa (10.67%) from already scarred uteruses. Stratification revealed a higher trend of the morbidity with the increase in number of previous caesarean sections.

**Conclusion:**

A significantly higher frequency of placenta Previa was found among patients coming to a tertiary care hospital with previously scarred uterus.

## INTRODUCTION

Placenta Previa is an obstetric complication that occurs in the second and third trimester of pregnancy. It may cause serious morbidity and mortality to the mother.^1^,^2^

It is a condition in which the placental tissue lies abnormally close to the internal cervical Os. Surgical disruption of the uterine cavity is a potential risk factor for placenta Previa and placental abruption. Approximately 10% cases placenta Previa are associated with placenta accrete.^3^-^5^

In United States, placenta Previa occurs in 0.3-0.5% of all pregnancies. The risk increases 1.5-5 folds with a history of caesarean delivery. With an increase number of deliveries, the risk can be as great as 10%. Although placenta Previa is relatively uncommon (incidence of 3 to 9 per 1000 pregnancies), it is regarded as one of the leading causes of uterine bleeding during the latter stages in gestation and has been recognized as an important determinant of maternal morbidity and adverse perinatal outcomes. It is a potentially life threatening condition that requires a multidisciplinary approach to management.^1^,^6^-^11^

The women at greatest risk of placenta Previa are those who have myometrial damage caused by a previous caesarean delivery with either anterior or posterior placenta Previa overlying the uterine scar. The value of making the diagnosis of placenta Previa before delivery is that it allows for multidisciplinary planning in an attempt to minimize potential maternal or neonatal morbidity and mortality.^5^,^11^-^13^

It classically presents as painless bleeding. Bleeding is thought to occur in association with the development of the lower uterine segment in the third trimester. Placental attachment is disrupted as this area gradually thins in preparation for the onset of labor. The diagnosis is usually established by ultrasonography and occasionally supplemented by magnetic resonance imaging.^14^,^15^

Accurate prenatal identification of affected pregnancies allows optimal management because timing and site of delivery, availability of blood products, and recruitment of a skilled anesthesia and surgical team can be arranged in advance.^13^,^16^,^17^ The study was conducted to determine the frequency of placenta Previa in patients coming to a tertiary care unit with previously scarred and non-scarred uterus.

## METHODS

A cross sectional survey was conducted for 12 months from 1st January 2012-31st December 2012 in the Lady Willingdon Hospital, Lahore. A non-probability, purpose sampling technique was adopted for enrolling the cases of placenta Previa. A total of 114 cases of placenta Previa fulfilling the criteria were enrolled to determine the frequency of placenta Previa in patients coming to a tertiary care unit with previous scarred and non-scarred uterus. During the study period, total LSCS performed in previously scarred uterus were 721, while in non scarred uterus were 645.

### Inclusion Criteria

Age 20-40 years, Patients with placenta Previa with scarred and non-scarred uterus. Singleton pregnancy and Gestational age 28 weeks and onwards,

### Exclusion Criteria

Primi gravidas, Second trimester bleeding and Scars other than C-section e.g. myomectomy.

### Data Collection

Patients presenting in emergency and outpatient department of unit-II of Lady Willingdon Hospital, Lahore were included in the study after fulfilling the inclusion and exclusion criteria’s. Detailed history was taken regarding age, parity, duration of gestation. All the information was collected through especially designed Performa.

## RESULTS

Age distribution of the patients was done, where in 30 patients were between 20-25 years of age group, 54 between 26-30 years, 26 between 31-35 years, and only 4 patients were between 36-40years.

Gestational age of the patients revealed 11 between 28-32 weeks, 23 between 33-36 weeks, and 80 between 36-40 weeks. (Table-I) Regarding gravidity, 67 patients were between G2-G4, 42 between G5-G7, and 5 were more than G7. (Table-II)

**Table-I:** Age of the patients with placenta Previa in previously scarred and non-scarred uterus.

Age (Years)	No.of patients	%age	Gestational age	No.of patients	%age
20-25	30	26.31%			
26-30	54	47.36%	28-32 weeks	11	9.64%
31-35	26	22.80%	33-36 weeks	23	20.17%
36-40	4	3.50%	36-40 weeks	80	70.17%
Total	114	100%		114	100%

**Table-II:** Regarding gravidity of Placenta Previa, types and frequency in 67 patients.

Gravidity	No.of patients (n=114)	%age
G2-G4	67	58.77
G5-G7	42	36.84
>G7	5	4.38
Placenta Previa in scarred uterus	77 patients	67.54%
Placenta Previa in non-scarred uterus	37 patient	32.45%

Type of Previa	No.of patients	%age
Major degree	88	77.19
Minor degree	26	22.80

No.of previous sections	No.of patients	%age
1	18	23.37
2	26	33.76
3	29	37.66
4	04	5.1

Stratification for placenta Previa according to previous caesarean sections was done which showed that out of 114 cases of placenta Previa, 18 had history of previous one LSCS, 26 had two LSCS, 29 had three LSCS, and 4 had previous four LSCS. The frequency of placenta Previa found in previously scarred uterus was 5.70%, while it was 10.67% in non scarred uterus.

In our study, 77 patients with placenta Previa had previous caesarean sections while 37 patients had previous vaginal deliveries. Degree of placenta Previa revealed 88 with major degree Previa and 26 with minor degree Previa.

## DISCUSSION

Placenta Previa can have serious adverse consequences for both mother and baby including an increased risk of maternal and neonatal mortality, fetal growth restriction and preterm delivery, antenatal and intrapartum hemorrhage, and women may require blood transfusions or even an emergency hysterectomy.^1^,^8^,^18^-^20^ The risk of placenta Previa is also reported to be higher among women with previous uterine surgery, including caesarean section.^21^,^22^

We carried out this study with the view that a number of women are delivered by caesarean sections in our setup on daily basis and surprisingly no published data is available regarding frequency of placenta Previa in previously scarred and non-scarred uterus, while the international data is also variant. Considering these issues, the results of the study may be helpful for the patients regarding awareness of frequency of placenta Previa in pregnancies followed by normal vaginal deliveries or followed by caesarean deliveries so that the obstetricians can manage these patients accordingly.^23^,^24^

In our study, out of 114 cases, most patients (47.36%) were between 26-30 yrs. age group, presented with gestational age between 36-40 weeks (70.17%), were mostly G2-4, while frequency of placenta Previa in non-scarred uterus was 32.45% (37 cases), and frequency in previously scarred uterus was 67.54%(77 cases). Major degree Previa was found in 88 cases(77.19%) and minor degree was in 26 cases(22.80%). Frequency of previous caesarean section was also recorded while stratification revealed a higher trend of the morbidity with the increase in number of previous caesarean sections.

During the study period, caesarean sections performed on the non-scarred uterus were 645 and among them 37 were carried out for placenta Previa (5.70%) while caesarean sections performed on previously scarred uterus were 721, and among them 77 were carried out for placenta Previa (10.67%).

Our findings are in accordance with Suknikhom W, Tannirandorn Y, who reported that previous uterine operations were found in the placenta Previa group more than the control group.^6^ In another study, Yazdani T found that out of 122 cases with previous history of C- section, placenta Previa was diagnosed in 19 cases(15.5%).^25^ Akram H found that 14 (23.3%) patients out of60 patients with placenta Previa had history of previous caesarean section.^11^

An association between placenta Previa and placental abruption with prior caesarean delivery is biological plausible.^20^ It is likely that a uterine low segment scar impairs placental attachment. Ligation of uterine vessels at the time of caesarean section may further increase the risk of damage to the endometrial and myometrial uterine lining, or both, which can predispose to a low implantation of the placenta in the uterus in the next pregnancy. It is speculated that the uterine muscle section during abdominal delivery interfered with its physiological stretching, and prevented or restricted the placenta moving away to the upper uterine segment in a subsequent pregnancy.^2^,^3^,^5^,^18^,^24^

However, we determined that caesarean section in previous pregnancy is moderately associated with placenta Previa in the following pregnancies. Clinicians might consider this information valuable when they counsel women during pregnancy.

## CONCLUSION

The results of the study conclude that a significantly higher frequency of placenta Previa was found among patients coming to a tertiary care hospital with previously scarred uterus.
